# Hepatitis B Serological Immunity and Exposure Among Blood Donors in Southern Croatia: A Cross-Sectional Study

**DOI:** 10.3390/vaccines13101027

**Published:** 2025-09-30

**Authors:** Admir Dilberovic, Mirela Radman-Livaja, Ivana Talic-Drlje, Ana Stanic, Marina Njire-Braticevic, Nikolina Tomicic, Jurica Arapovic

**Affiliations:** 1 Department of Transfusion Medicine, Dubrovnik General Hospital, 20000 Dubrovnik, Croatia; 2 Faculty of Health Studies, University of Mostar, 88000 Mostar, Bosnia and Herzegovina; 3 Department of Transfusion Medicine, University Hospital Split, 21000 Split, Croatia; 4 Faculty of Health Sciences, University of Split, 21000 Split, Croatia; 5 Department of Transfusion Medicine, University Hospital Mostar, 88000 Mostar, Bosnia and Herzegovina; 6 Faculty of Medicine, University of Mostar, 88000 Mostar, Bosnia and Herzegovina; 7 Department of Medical Biochemistry Laboratory, Karlovac General Hospital, 47000 Karlovac, Croatia; 8 Department of Laboratory Diagnostics, Dubrovnik General Hospital, 20000 Dubrovnik, Croatia; 9 Center for Disease Control and Geohealth Studies, Academy of Sciences and Arts of Bosnia and Herzegovina, 71000 Sarajevo, Bosnia and Herzegovina

**Keywords:** hepatitis B, anti-HBc, anti-HBs, seroprevalence, blood donors, transfusion safety, vaccination

## Abstract

**Background/Objectives:** Hepatitis B virus (HBV) remains a persistent challenge for transfusion safety. Although testing for hepatitis B surface antigen (HBsAg) and nucleic acid testing (NAT) reduces transmission risk, antibodies to hepatitis B core antigen (anti-HBc) and antibodies to hepatitis B surface antigen (anti-HBs) provide additional insight into past infection and vaccine-induced immunity. We aimed to determine their seroprevalence among blood donors in southern Croatia and assess associations with age, occupation, and time since vaccination. **Methods:** This cross-sectional study was conducted between February and November 2024 at two regional transfusion centers in southern Croatia. A total of 1008 voluntary blood donors, all HBsAg- and NAT-negative, were tested for anti-HBc and anti-HBs using chemiluminescent microparticle immunoassay. Demographic and vaccination data were collected through verified medical records. **Results:** Anti-HBc was detected in 0.5% of donors, exclusively among the unvaccinated. Protective anti-HBs levels were found in 38.1% overall and 70.6% of vaccinated donors, with significant declines by age and more than 15 years post-vaccination (*p* = 0.024). Healthcare workers showed higher seroprotection than non-healthcare donors (67.0% vs. 35.1%; *p* < 0.001), although one-third still lacked protective levels. **Conclusions:** HBV exposure was rare, but waning vaccine-induced immunity was evident, with protective anti-HBs levels in 70.6% of vaccinated donors, declining with age and time since vaccination. These findings highlight the need for periodic monitoring of anti-HBs and targeted booster strategies, especially in older and occupationally exposed groups. HBsAg and NAT provide a high level of transfusion safety, while the role of routine anti-HBc testing in this low-endemic context should be carefully evaluated in view of its potential benefits and drawbacks. Donor-based surveillance is a valuable tool for evaluating long-term vaccine effectiveness and guiding public health policy.

## 1. Introduction

Hepatitis B virus (HBV) remains a significant global public health challenge despite the availability of effective vaccines and advanced screening methods [1]. The World Health Organization (WHO) aims to eliminate HBV as a public health threat by 2030 through expanded vaccination and improved blood safety measures [2]. In transfusion medicine, hepatitis B has historically been one of the most significant transfusion-transmitted infections (TTIs) [3], and careful donor selection with serological testing remains critical [4]. Modern donor screening with hepatitis B surface antigen (HBsAg) and nucleic acid testing (NAT) has markedly reduced transmission risk, yet residual risk persists due to the diagnostic “window period” and occult HBV infection (OBI) [5,6,7]. In Croatia, the estimated residual risk of HBV transmission between 2013 and 2016 was 1 in 36,900 donations, despite existing testing protocols [8]. Residual risk is mainly associated with donations that are negative for HBsAg and/or HBV DNA, particularly when collected during the early acute or late phases of infection. The likelihood of detecting such cases depends on the screening strategy applied and the performance of serological and molecular assays [3].

The role of routine antibodies to hepatitis B core antigen (anti-HBc) testing in low-endemic, NAT-screened settings remains debated due to its limited diagnostic yield and cost-effectiveness. Nevertheless, anti-HBc is a reliable marker of past HBV infection, and more than 80% of confirmed OBI cases are anti-HBc positive. The detection of HBV infection in the absence of HBsAg, with the presence of anti-HBc and/or antibodies to hepatitis B surface antigen (anti-HBs), highlights the added value of serological testing in improving blood donation safety protocols [7]. Serological data consistently show that anti-HBc prevalence increases with age, reflecting higher exposure before the introduction of universal vaccination [9,10]. While countries such as Germany and the Netherlands have incorporated anti-HBc screening into donor protocols, Croatia currently does not mandate this testing. Donors with a history of HBV infection are permanently deferred, and routine screening remains limited to HBsAg and NAT. Notably, since 2013 Croatia has applied individual donation (ID)-NAT to all donations, reflecting the adoption of the most advanced and sensitive molecular screening approach [7].

Vaccination is the most effective strategy for preventing HBV infection. Following WHO recommendations in 1992, many countries adopted universal hepatitis B immunization [11]. In Croatia, systematic HBV vaccination was introduced in 1999 for school-aged children, administered as a three-dose intramuscular schedule in the 6th grade using a recombinant HBsAg vaccine. In 2007, the program was revised to target infants, with vaccination administered according to the national three-dose schedule. According to national immunization reports, coverage in these cohorts has consistently exceeded 90%, ensuring high protection in the younger population. However, systematic data on vaccination coverage in older adults are not available. Outside these programs, vaccination is recommended for defined risk groups, including healthcare workers (HCWs), household contacts of HBsAg-positive individuals, patients on hemodialysis, and those with selected comorbidities [12]. As a result, younger generations are expected to demonstrate higher vaccine-induced protection compared to older cohorts, who may remain susceptible or rely on naturally acquired immunity. While more than 90% of healthy vaccine recipients initially develop protective levels of anti-HBs, concentrations decline over time [13,14]. The durability of vaccine-induced immunity is still debated, with some evidence suggesting persistence of immune memory despite waning antibody levels, while other studies support booster vaccination in selected groups such as HCWs. A threshold of ≥10 mIU/mL anti-HBs is generally considered protective [15,16].

Blood donors represent a valuable sentinel population for monitoring HBV exposure and immunity. They are routinely screened for transfusion safety, are generally healthy, and provide a large, well-characterized cohort for seroepidemiological studies. Assessing anti-HBc and anti-HBs prevalence among blood donors not only informs transfusion safety but also contributes to public health planning by evaluating vaccination program outcomes and identifying groups at risk of declining immunity, particularly HCWs [17].

Importantly, while several Croatian studies have reported trends in anti-HBc prevalence, anti-HBs seroprevalence has never been systematically investigated among blood donors at the national level. Similarly, data remain scarce in neighboring countries such as Austria, underscoring the importance of regional studies to evaluate whether past vaccination initiatives have provided durable population protection [18]. Previous studies in Croatia have demonstrated a steady decline in anti-HBc prevalence among blood donors over the past two decades, reflecting the impact of vaccination and improved safety protocols [19,20]. To date, region-specific data from southern Croatia have not been reported, and we provide them in this study.

We therefore aimed to assess the seroprevalence of anti-HBc and the proportion of donors with protective anti-HBs levels in a large donor cohort from southern Croatia, and to analyze their associations with age, occupation, and time since vaccination. By addressing these gaps, this study provides novel epidemiological data relevant for transfusion safety, vaccination policy, and the evaluation of long-term vaccine-induced protection, including considerations for booster strategies in high-risk groups such as HCWs.

## 2. Materials and Methods

This cross-sectional study was conducted between February and November 2024 at two transfusion centers in southern Croatia: the University Hospital Split and the General Hospital Dubrovnik. These centers jointly serve the Split–Dalmatia, Dubrovnik–Neretva, and Šibenik–Knin counties, covering a donor catchment area of approximately 687,000 inhabitants. Together, they collect blood from an estimated 12,000 donors annually. A total of 1008 voluntary blood donors were enrolled. Donors were classified as first-time or repeat/return donors according to national blood service definitions. To reflect the introduction of the mandatory school-age vaccination program in 1999, donors were further stratified by age (≤37 vs. ≥38 years), with the younger group most likely vaccinated under the mandate and the older group largely unvaccinated except by indication (e.g., healthcare workers). Eligible participants were aged 18–65 years, Croatian citizens, and provided written informed consent. Each participant received standardized information regarding the study’s purpose, procedures, and potential risks and benefits. Donor eligibility followed national regulations for routine blood donation. Sociodemographic characteristics and vaccination history were obtained through structured interviews and verified through vaccination records where available. In cases of incomplete information, regional public health databases were consulted with participant permission. Venous blood samples were collected according to standard phlebotomy procedures. Serum obtained during routine donation testing was used for this study to avoid additional burden on participants. Samples were processed and stored at −25 °C and transferred under controlled conditions to the Department of Transfusion Medicine, Dubrovnik General Hospital. All blood donations were screened for HBsAg using the ARCHITECT HBsAg Qualitative II assay (Abbott Diagnostics, Wiesbaden, Germany), following the manufacturer’s instructions. Serological testing for anti-HBc and anti-HBs was performed using chemiluminescent microparticle immunoassay (CMIA) on the ARCHITECT i2000SR analyzer (Abbott Diagnostics, Abbott Park, IL, USA). According to the manufacturer, the assays demonstrated 100% sensitivity and >99.5% specificity for anti-HBc, and 97.5% sensitivity and 99.7% specificity for anti-HBs [21,22]. To ensure analytical validity, a subset of samples was selected by simple randomization, with equal probability of selection for each sample, and retested using chemiluminescent immunoassay (CLIA) on the Siemens ADVIA Centaur CP (Siemens Healthcare GmbH, Erlangen, Germany) as part of the analyzer verification performed in accordance with the Clinical and Laboratory Standards Institute (CLSI) EP15-A3 guidelines. Anti-HBc was interpreted qualitatively, while anti-HBs concentrations were reported in mIU/mL, with ≥10 mIU/mL defined as protective [15,16].

ID-NAT for HBV DNA was performed centrally at the Croatian Institute of Transfusion Medicine in Zagreb, the national reference laboratory since the implementation of mandatory NAT in 2013. NAT was conducted using the Procleix Ultrio Plus assay (Grifols, Barcelona, Spain) on the Procleix platform, according to the manufacturer’s recommendations [8].

Data analysis was performed using IBM SPSS Statistics v26. Categorical variables were compared with Pearson’s chi-square or Fisher’s exact test where appropriate. Normality of continuous variables was tested with Kolmogorov–Smirnov and Shapiro–Wilk tests. As most distributions were non-normal, non-parametric tests (Mann–Whitney U and Kruskal–Wallis) were applied. A two-sided *p*-value < 0.05 was considered statistically significant. An a priori power analysis was conducted using G*Power v3.1.9.6 (University of Kiel, Kiel, Germany). With an assumed effect size of 0.2, α = 0.05, and 80% power, the minimum required sample size was calculated at 788 participants.

## 3. Results

A total of 1008 donors were included, of whom 73 (7.2%) were first-time and 935 (92.8%) were repeat or return donors. The mean age was 38.2 ± 10.8 years (range: 18–65). Most participants were male (*n* = 801; 79.5%) and 88 (8.7%) were HCWs. In accordance with standard HBV screening protocols, all donors were initially tested for HBsAg and NAT, and all results were negative. To ensure accurate estimation of seroprevalence within the donor population, additional serological testing for anti-HBc and anti-HBs was performed on all samples, regardless of age or vaccination history. Anti-HBc was detected in 5 donors (0.5%), all of whom were unvaccinated. Among the 73 first-time donors, 1 (1.4%) was anti-HBc positive and 72 (98.6%) were negative, while the remaining 4 positive cases were observed among repeat or return donors (4/935, 0.4%). The difference according to vaccination status was statistically significant (Fisher’s exact test, *p* = 0.022). None of the anti-HBc-positive individuals had detectable HBV DNA, indicating the absence of OBI (Table 1).

Among all participants, 537 donors (53.3%) had documented HBV vaccination, while 471 (46.7%) had no vaccination record (Table 1). Within the vaccinated subgroup, 379 donors (70.6%) exhibited protective anti-HBs levels. Notably, all five anti-HBc-positive donors—who were unvaccinated—also had protective anti-HBs levels, suggesting past resolved HBV infection. In total, 384 donors (38.1%) demonstrated serological evidence of protective immunity (Table 2).

**Figure 1:**
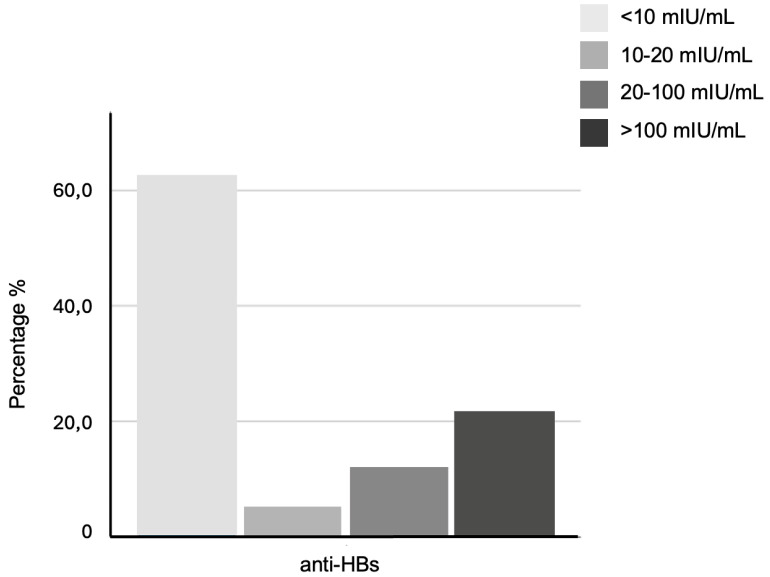
Anti-HBs levels in all study donors (N = 1008), grouped into four categories (<10, 10–20, 20–100, >100 mIU/mL). Bars show the percentage (%) of donors in each category.

Anti-HBs levels were categorized into four ranges (<10, 10−20, 20−100, and >100 mIU/mL) to assess immunity distribution, identify donors near the protective threshold, and examine relationship with age and time since vaccination (Figure 1).

A statistically significant association was found between donor age and anti-HBs levels (*p* < 0.001). Donors with non-protective levels (<10 mIU/mL) were predominantly older (38–65 years), whereas a strong immune response (>100 mIU/mL) was observed mainly in younger donors (18–37 years; 41.4% vs. 2.7% in older donors). Notably, all older donors with protective anti-HBs levels were HCWs, suggesting targeted adult immunization through occupational health programs (Table 3). No significant correlation was observed between anti-HBs levels and gender (*p* = 0.186), with comparable distributions in males and females. In contrast, vaccination status strongly correlated with seroprotection (*p* < 0.001). Among vaccinated donors, 39.9% achieved strong immune response, while almost all unvaccinated donors (98.9%) were distributed in the <10 mIU/mL group. A subgroup of 44 vaccinated donors (9%) exhibited anti-HBs levels near the protective threshold (10–20 mIU/mL), suggesting potential waning immunity (Table 3). Time since vaccination also influenced antibody persistence (*p* = 0.024), with higher portion of strong immune responses among those vaccinated within the past 15 years compared to those vaccinated earlier (45.7% vs. 36.5%) (Table 3).

**Figure 2:**
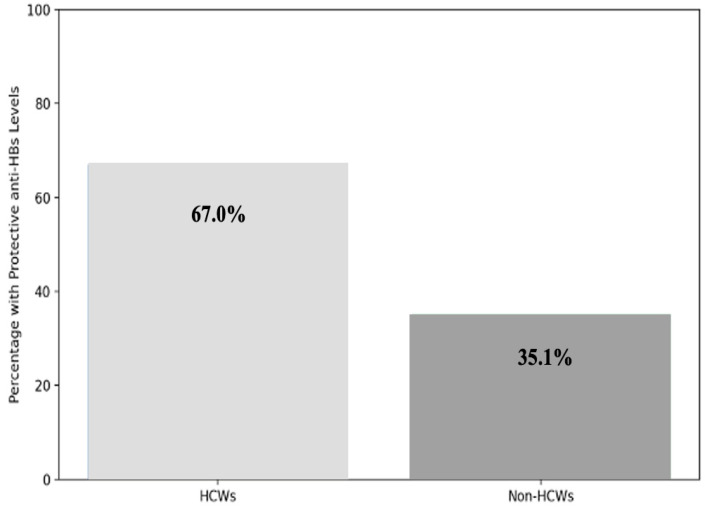
Comparison of protective anti-HBs levels in HCWs and non-HCWs.

Protective immunity differed by occupation. HCWs had significantly higher seroprotection than non-HCWs (67.0% vs. 35.1%; *p* < 0.001). However, one-third of HCWs still lacked protective levels, despite occupational vaccination policies (Figure 2).

The durability of vaccine-induced immunity was evaluated by comparing donors vaccinated <15 years ago with those vaccinated > 15 years ago. Among vaccinated donors (*n* = 537), those vaccinated within the last 15 years exhibited significantly higher rates of seroprotection (152/197, 77.2%) compared to those vaccinated earlier (227/340, 66.8%) (*p* = 0.024). Although a progressive decline in antibody concentrations and seroprotection was observed, a substantial proportion of donors maintained protective levels beyond 15 years after vaccination.

## 4. Discussion

This study provides the first comprehensive data on hepatitis B serological immunity and exposure among blood donors in southern Croatia, and importantly, the first assessment of anti-HBs seroprevalence in any donor population in Croatia. Our findings demonstrate a very low prevalence of past HBV infection in this low-endemic, NAT-screened setting, with anti-HBc reactivity confined to unvaccinated donors. At the same time, we observed waning vaccine-induced protection with increasing age and time since vaccination, underscoring the need to evaluate long-term durability of HBV immunity and consider targeted interventions. The overall anti-HBc seroprevalence in our cohort was 0.5%, with all positive cases found in unvaccinated donors. Anti-HBc seroprevalence was slightly higher among first-time donors (1/73, 1.4%) than repeat donors (4/935, 0.4%), supporting the view that first-time donors may carry a higher risk. However, some studies have reported higher prevalence among older repeat donors, most likely due to age and lack of vaccination [6,8].

None were HBV DNA-positive, and no cases of OBI were identified. These results are consistent with previously reported trends in Croatia. Miletic et al. reported a decline in anti-HBc prevalence from 5.24% in 2004 to 1.32% in 2017 among Croatian blood donors [19], and Samardzija et al. found 1.5% in eastern Croatia [20]. Similarly low prevalence rates are reported in Switzerland [23] and in countries with established vaccination programs and robust donor screening protocols, such as Germany and the Netherlands [7]. Outside Europe, prevalence varies significantly. A large Canadian study reported 1.37% among over 1.5 million blood donors [24], while significantly higher rates occur in Iran and Pakistan (6–8%) [25]. In Libya, estimates range from 10% to 15.6%, depending on test methodology and regional sampling [26,27,28,29]. A study from Iran by Karimi et al. found anti-HBc positivity in 4.9% of HBsAg-negative donors [6], while a similar study in Egypt reported a prevalence of 17.2% [30]. These comparisons highlight the success of Croatia’s national vaccination strategy in reducing HBV exposure.

From a transfusion perspective, OBI yields vary between countries due to differences in baseline HBV epidemiology, donor population structure (age distribution and the portion of first-time vs. repeat donors) and screening portfolios. Programs that include NAT and/or anti-HBc screening detect more OBI than those relying on HBsAg alone, and assay selection is shaped by resource availability and national policy [3]. Notably, a study from Croatia reported that OBI was more frequently detected in older repeat blood donors, with nearly all cases (98%) being anti-HBc positive [8]. Although anti-HBc is a sensitive marker of past HBV infection, its diagnostic value is reduced by the high analytical sensitivity of current assays, which can yield false-positive results and result in unnecessary donor deferrals [31]. This concern is particularly important in countries with smaller donor populations, such as Croatia, where maintaining a sufficient blood supply is already challenging. A study from Croatia estimated that deferring all anti-HBc positive donors would result in the exclusion of at least 1.32% of blood donors [8]. Nevertheless, the quality and safety of blood products remain the primary priority, and any screening strategy must balance these requirements with the need to maintain an adequate donor base [2].

Analysis of anti-HBs levels revealed patterns highly relevant for vaccination policy. Protective immunity was present in 70.6% of vaccinated donors, but only in 38.1% overall. Importantly, this protection should be interpreted with caution. Although 70% of vaccinated donors had anti-HBs ≥ 10 mIU/mL, almost 9% were in the borderline 10–20 mIU/mL range, which reduces the proportion of satisfactorily protected vaccinated donors to closer to 60%. From a transfusion perspective, however, blood safety does not depend on donor anti-HBs levels but on rigorous HBsAg and NAT screening, which in our cohort identified no HBV DNA-positive donations or OBI. Younger donors demonstrated higher antibody levels, consistent with the expansion of universal vaccination to schoolchildren in 1999. Conversely, older donors, who were largely unvaccinated or vaccinated decades ago, showed lower levels of seroprotection, consistent with findings from Serbia, where vaccinated individuals aged 1–19 had 80.7% seroprevalence compared to 53.9% overall [10]. Comparable rates were reported in Tyrol, Austria (51.4%) [18], whereas lower rates were observed in Turkey (27.4%) [32]. These regional differences may reflect variations in vaccine coverage, timing of program implementation, and inclusion of high-risk groups. Comparisons with earlier studies conducted 10 to 15 years ago in both donor populations and the general population reinforce the impact of vaccination. Anti-HBs seroprevalence was 15.9% in Egypt [33], 23.8% in Italy [34], and ranged from 2.0% to 12.7% in Nigeria [35,36].

A relevant Croatian study from 2011 involving patients attending routine check-ups reported an anti-HBs-only prevalence of 24.4%, notably lower than in our vaccinated cohort [17]. We found no significant gender-based differences, suggesting similar vaccine coverage and responsiveness in males and females. However, as women account for only about 16% of the Croatian donor population, this limited representation may have reduced the ability to detect subtle gender-related differences in our cohort [37]. A study from neighboring Bosnia and Herzegovina also documented a sharp decline in HBV prevalence among blood donors following the implementation of mandatory hepatitis B vaccination and enhanced pre-donation questionnaires [38].

HCWs in our cohort had significantly higher protection rates than non-HCWs (67.0% vs. 35.1%), likely reflecting workplace vaccination. However, over one-third lacked protective levels, a concerning finding given occupational risk. In Iran, Joukar et al. reported 88.9% protection among HCWs, but such comparisons should consider cohort age, vaccination timing, and local epidemiology [39]. Sahana et al. observed protection in 94.1% of individuals within 5 years of vaccination, decreasing to 79.7% after 6–10 years and 72.7% beyond 10 years [40]. Ippoliti et al. recently reported that only 55% of medical students in Rome had protective anti-HBs levels, with higher anti-HBs levels among those vaccinated in the first year of life and a marked decline associated with longer time since vaccination [41]. This highlights the importance of monitoring post-vaccination immunity and considering booster strategies for occupationally exposed individuals reinforcing the need for tailored policies that go beyond universal childhood vaccination. Taken together, our findings emphasize the dual value of blood donor studies: they provide epidemiological surveillance of past HBV exposure through anti-HBc and simultaneously serve as a practical tool for monitoring the effectiveness and durability of vaccination programs via anti-HBs testing. The absence of systematic data on anti-HBs seroprevalence among blood donors in Croatia has limited evaluation of the country’s vaccination strategy. Our study therefore fills an important gap and provides baseline data for future monitoring in line with WHO’s HBV elimination goals.

A key limitation of our study is the inability to identify immunological non-responders—those who do not develop protective anti-HBs levels despite full vaccination. This condition, affecting approximately 5–10% of vaccinated individuals, is more common in older adults and those with chronic conditions or immunosuppression, but is likely rare among young, healthy blood donors [42]. The presence of undetected non-responders may slightly influence our seroprevalence estimates. Another limitation of our study is the inability to distinguish between primary non-responders and individuals with waning immunity, as post-vaccination anti-HBs levels were not available. Additionally, we cannot exclude the contribution of immune memory in individuals with low antibody levels, which may provide protection upon re-exposure [43]. Nonetheless, our cross-sectional design offers valuable insights into population-level immunity patterns and their determinants.

## 5. Conclusions

HBV exposure was rare among NAT-screened blood donors in southern Croatia, with anti-HBc detected only in unvaccinated individuals. In contrast, protective anti-HBs levels were observed in 70.6% of vaccinated donors but only 38.1% overall, declining significantly with age and more than 15 years after vaccination. These findings indicate that while Croatia’s vaccination program has been effective in younger cohorts, waning immunity remains a concern, particularly for older and occupationally exposed donors. Post-vaccination monitoring and targeted booster strategies may therefore be warranted, especially among healthcare workers. HBsAg and NAT provide a high level of transfusion safety, while the role of routine anti-HBc testing in this low-endemic context should be carefully evaluated in view of its potential benefits and drawbacks. These findings underscore the role of donor-based surveillance in evaluating vaccination programs and shaping public health policies to ensure long-term HBV protection.

## Figures and Tables

**Table 1:** Association between vaccination status and anti-HBc seroreactivity among blood donors.

Vaccination Status	Anti-HBc Negative, *n*/N (%)	Anti-HBc Positive *n*/N (%)	Pearson Chi-Square (*p*-Value)	Fisher’s Exact Test (*p*-Value)
Yes	537/537	0/537	0.017	0.022
	(100.0%)	(0.0%)		
No	466/471	5/471		
	(98.9%)	(1.1%)		
Total	1003/1008 (99.5%)	5/1008 (0.5%)		

Data are presented as *n*/N (%). *n* = number of donors in the subgroup; N = total number of donors in that category. Statistical significance was assessed using both the χ^2^ test (*p* = 0.017) and Fisher’s exact test (*p* = 0.022), the latter applied due to the small number of anti-HBc-positive donors (*n* = 5).

**Table 2:** Distribution of protective anti-HBs levels by vaccination status.

Vaccination Status	Number of Donors	Donors with >10 mIU/mL (*n*, %)	Interpretation
Vaccinated	537	379 (70.6%)	Vaccine-induced immunity
Unvaccinated	471	5 (1.1%)	Resolved infection
Total	1008	384 (38.1%)	Overall protective anti-HBs levels

**Table 3:** Distribution of anti-HBs levels according to age, gender, vaccination status, time since vaccination and healthcare worker status.

Variable	Age Group		Gender		Vaccination Status		Time Since Vaccination (Years)		Healthcare Worker	
	18–37 (*n* = 490)	38–65 (*n* = 518)	Male (*n* = 801)	Female (*n* = 207)	Yes (*n* = 537)	No (*n* = 471)	<15 (*n* = 197)	>15 (*n* = 340)	Yes (*n* = 88)	No (*n* = 920)
Anti-HBs < 10 (*n*, %)	135 (27.6%)	489 (94.4%)	503 (62.8%)	121 (58.5%)	158 (29.4%)	466 (98.9%)	45 (22.8%)	113 (33.2%)	29 (33.0%)	595 (64.7%)
Anti-HBs 10–20 (*n*, %)	44 (9.0%)	4 (0.8%)	41 (5.1%)	7 (3.4%)	48 (8.9%)	0 (0.0%)	22 (11.2%)	26 (7.6%)	7 (8.0%)	41 (4.5%)
Anti-HBs 20–100 (*n*, %)	108 (22.0%)	11 (2.1%)	87 (10.9%)	32 (15.5%)	117 (21.8%)	2 (0.4%)	40 (20.3%)	77 (22.6%)	17 (19.3%)	102 (11.1%)
Anti-HBs > 100 (*n*, %)	203 (41.4%)	14 (2.7%)	170 (21.2%)	47 (22.7%)	214 (39.9%)	3 (0.6%)	90 (45.7%)	124 (36.5%)	35 (39.8%)	182 (19.8%)
Total	490 (100%)	518 (100%)	801 (100%)	207 (100%)	537 (100%)	471 (100%)	197 (100%)	340 (100%)	88 (100%)	920 (100%)
*p*-value (Pearson Chi-Square)	<0.001 *		0.186		<0.001 *		0.024 *		<0.001 *	

* Statistically significant difference: *p* < 0.05; anti-HBs values are expressed in mIU/mL; percentages are rounded and may not total 100%.

## Data Availability

Participant-level data from the blood donor cohort cannot be shared due to regulatory restrictions. Aggregated data supporting the findings of this study are available from the corresponding author upon reasonable request.

## Abbreviations

The following abbreviations are used in this manuscript:

HBV	Hepatitis B virus
HBsAg	Hepatitis B surface antigen
NAT	Nucleic acid testing
OBI	Occult hepatitis B infection
TTI	Transfusion-transmitted infection
anti-HBc	Antibody to hepatitis B core antigen
anti-HBs	Antibody to hepatitis B surface antigen
HCWs	Healthcare workers
